# 吉非替尼对肺癌细胞株H358放疗敏感性的影响及其机制

**DOI:** 10.3779/j.issn.1009-3419.2011.11.02

**Published:** 2011-11-20

**Authors:** 洁 邓, 亮 庄, 元 陈

**Affiliations:** 430030 武汉，华中科技大学同济医学院附属同济医院肿瘤中心 Cancer Center, Tongji Medical College, Huazhong University of Science and Technology, Wuhan 430030, China

**Keywords:** 表皮生长因子受体, 吉非替尼, 肺肿瘤, H358细胞, γ-H2AX, EGFR, Gefitinib, Lung neoplasmas, H358, γ-H2AX

## Abstract

**背景与目的:**

表皮生长因子受体（epidermal growth factor receptor, EGFR）是决定放疗效应的重要因素，它的过表达或激活常与包括非小细胞肺癌在内的肿瘤放疗抵抗相关，因而阻断EGFR的信号通路是增强放疗敏感性很有潜力的治疗策略。本研究旨在观察小分子EGFR酪氨酸激酶抑制剂吉非替尼与放疗联合是否有提高非小细胞肺癌细胞株H358放疗敏感性的作用以及探索其分子机制。

**方法:**

将非小细胞肺癌细胞株H358分为X线组和X线+吉非替尼组，前者采用单纯X线照射，后者经1 μmol/L吉非替尼作用24 h后再行X线照射。克隆形成实验比较两组细胞放射敏感性，免疫荧光激光共聚焦显微镜观察X线照射后各时间点细胞核中磷酸化γ-H2AX及EGFR焦点在细胞中的定位情况，Western blot法检测放疗后核蛋白中EGFR的表达。

**结果:**

克隆形成实验中X线+吉非替尼组各个剂量点的细胞存活率均少于X线组，可见X线+吉非替尼组对放疗更敏感。免疫荧光激光共聚焦显示，X线+吉非替尼组比X线组各时段细胞核内γ-H2AX焦点数增加，持续的时间也更长。EGFR免疫荧光及Western blot结果显示，X线组EGFR在放疗后1 h内入核，而X线+吉非替尼组EGFR不在核内表达，仍位于细胞浆内。对Western blot

**结果:**

用SPSS 13.0进行统计学分析，其差异有统计学意义（*P*=0.042）。

**结论:**

吉非替尼可能是通过抑制EGFR放疗后入核进行损伤后DNA双链断裂修复，而起到对NSCLC细胞株H358放疗增敏的作用。

## Abstract (en)

**Background and objective:**

The epidermal growth factor receptor (EGFR) is an important determinant of radioresponse, the elevated expression and activity of which frequently correlates with radioresistance in several cancers, including non-small-cell lung carcinoma (NSCLC). The molecular blockade of EGFR signaling is a promising therapeutic strategy for the enhancement of the cytotoxic effects of radiotherapy. The aims of the present study are to observe whether gefitinib, a selective EGFR tyrosine kinase inhibitor, can radiosensitize the NSCLC H358 cell line and to investigate the mechanism by which this drug restores the radiosensitivity of NSCLC cells.

**Methods:**

NSCLC cell line H358 was divided into two groups, namely the X-ray and the gefitinib-interfering groups. The former was irradiated using X-ray only, and the latter was treated with 1 μmol/L gefitinib 24 h before irradiation under the same conditions. The cells were tested using the clonogenic cell survival assay to identify the radiosensitivity of both groups. Immunostaining for confocal microscopy was used to observe nuclear γ-H2AX repair and EGFR foci after irradiation. Nuclear EGFR expression was detected using Western blot after radiotherapy.

**Results:**

In the clonogenic cell survival assay, the survival fraction in the gefitinib-interfering group was lower than that in the X-ray group at different doses. Surviving fraction of 2 Gy (SF2) were 0.000, 865 and 0.011, 1 for the gefitinib-interfering and the X-ray groups, respectively. Sensivive enhancement ratio (SER) was 2.815. Immunostaining for confocal microscopy suggested that more nuclear γ-H2AX foci are present in the gefitinib-interfering group than in the X-ray group. The nuclear γ-H2AX foci also stayed longer in the gefitinib-interfering group. EGFR translocated into the nucleus within 1 h in X-ray group, but stayed in the cytoplasma in the gefitinib-interfering group. Western blot was tested using SPSS 13.0, *P*=0.042.

**Conclusion:**

Gefitinib, at the cellular level, radiosensitizes EGFR with NSCLC H358 by blocking EGFR nuclear translocation as one of its mechanisms.

肺癌是当今世界上对人类健康和生命危害最大的恶性肿瘤之一，其中非小细胞肺癌（non-small cell lung cancer, NSCLC）占所有肺癌病例85%以上^[1]^。表皮生长因子受体（epidermal growth factor receptor, EGFR）是分子量为170 kDa的跨膜受体，在大多数上皮来源的肿瘤中表达增高，包括NSCLC^[2]^。放疗作为NSCLC的重要治疗手段，可直接或间接作用于DNA，其中DNA双链断裂（DNA double strand break, DSB）是最致命的^[3]^，但仍有一部分NSCLC对放疗不敏感。野生型EGFR可在放疗后1 h-4 h内入核，进行DNA损伤的修复而具有放疗保护的作用; 而NSCLC突变型*EGFR*不能入核修复，对放疗相对敏感^[3-5]^。吉非替尼和厄洛替尼可抑制EGFR胞内端酪氨酸激酶，阻止下游信号传导，达到抑制肿瘤细胞增殖，促进肿瘤细胞凋亡^[6]^。本实验旨在研究吉非替尼对NSCLC细胞株H358是否有放疗增敏作用及其机制。

## 1 材料与方法

### 1.1 材料

NCI-H358为人NSCLC细胞株，购自中国科学院细胞库，EGFR野生型，在基因水平上扩增，对吉非替尼敏感。主要试剂包括：吉非替尼（为阿斯利康公司赠予），称重研磨溶于DMSO，终浓度为10 mmol/L，分装后-20 ℃保存备用。EGFR（14C8）：sc-81450（Santa Cruz公司），Anti-phospho-Histone H2A.X（Ser139），clone JBW301（Millipore公司），Lamin B1 polyclonal antibody（Biovision公司），TRITC-II抗（Invitrogen公司），HRP-II抗（Antgene公司），BCA蛋白浓度检测分析试剂盒（Thermo公司），细胞核浆蛋白分离提取试剂盒（Thermo公司），ECL化学发光试剂盒（GE Healthcare公司），Complete, EDTA-free protease inhibitor cocktail tablets（Roche公司）。主要仪器包括：医用直线加速器（瑞典ELEKTA-precise），激光共聚焦显微镜（日本Olympus公司），凝胶成像灰度定量分析系统（美国Bio-Rad公司）。

### 1.2 实验分组及照射方法

将H358细胞分为X线组和X线+吉非替尼组，前者进行单纯X线照射，后者为H358细胞在1 μg/mL吉非替尼作用24 h后进行再进行X线照射。H358细胞照射采用同济医院医用直线加速器机架角180度，源皮距100 cm，6 MV X线等中心照射，平皿、细胞培养瓶或24孔板下垫1 cm厚有机玻璃板。

### 1.3 细胞培养

细胞培养采用含RPMI-1640培养液，10%胎牛血清，10, 000单位/mL青链霉素的培养基，于37 ℃，5%CO_2_培养箱培养，隔天换液，细胞传代每4-5天一次。

### 1.4 克隆形成实验

消化细胞，计数后按不同照射剂量接种合适细胞（0 Gy、2 Gy、4 Gy、6 Gy、8 Gy分别接种的细胞数为200、5, 000、10, 000、20, 000、40, 000）到60 mm直径的平皿，两组细胞每个剂量设3个副孔。培养贴壁后实验组加含吉非替尼浓度为1 μmol/L的培养液，对照组只换液，药物作用24 h后分别进行0 Gy、2 Gy、4 Gy、6 Gy、8 Gy X线照射，重新换培养基继续培养10 d-14 d。形成克隆后，结晶紫染液染色20 min，自来水充分冲洗后晾干，显微镜下计数每一剂量下含细胞数大于50个的细胞克隆数。以0 Gy组计算克隆形成效率（plating efficicy, PE），计算各个剂量下的细胞存活率（survival fraction, SF），PE=（0 Gy剂量下集落数/细胞接种数）^*^100%，SF=某一剂量照射组细胞形成的克隆数/（该组细胞种植数×PE），用Excel拟合剂量生存曲线，SPSS 13.0软件利用单靶多击模型计算2 Gy的克隆形成率（surviving fraction of 2 Gy, SF2）、平均致死剂量（mean lethal does, D0）、准阈剂量（quasi-threshould does, Dq）、放射增敏比（sensitive enhancement ratio, SER）值。

### 1.5 免疫荧光

胰酶消化对数生长期的细胞，细胞计数板计数，稀释至1×10^5^/ml密度，接种约2, 000/孔细胞至24孔板，使细胞在盖玻片上贴壁生长，以4 Gy X线照射，X线+吉非替尼组以1 μmol/L吉非替尼提前24 h预处理。后于以PBS洗，4%多聚甲醛于放疗后不同时间（γ-H2AX分别于放疗后0 min，10 min，30 min，1 h，3 h，6 h，12 h，24 h）固定，EGFR于放疗后0 min，15 min，30 min，45 min，60 min，3 h）固定细胞15 min，PBS洗3遍，0.2% Triton X-100室温作用15 mim，PBS洗3遍，5%山羊封闭血清室温孵育1 h，加I抗1:100（γ-H2AX, EGFR）、4 ℃过夜，PBS洗3遍，TRITC-II抗1:50、37 ℃避光孵育50 min，PBS洗，1 μg/mL Hoechst33342避光孵育15 min，PBS洗，甘油封片，固定至载玻片上，避光放置，激光共聚焦显微镜观察照相。

### 1.6 免疫印记

以4 Gy X线照射两组生长至铺满瓶底80%-90%的细胞，X线+吉非替尼组以1 μmol/L吉非替尼提前24 h预处理，以放疗后不同时间（0 min, 15 min, 45 min, 60 min）胰酶消化，离心收集细胞，提取细胞核蛋白，BCA法测各组核蛋白浓度。制备5%浓缩胶，8%分离胶，以60 μg蛋白上样，电泳，275 mA电流转至PVDF膜140 min，5%脱脂奶粉室温封闭90 min，EGFR及Laminb1以1:1, 000稀释4 ℃孵育过夜，PBS洗，HRP-II抗以1:5, 000稀释室温孵育2 h，PBS洗，ECL室温作用5 min后稍吸干，于暗室胶片曝光。扫描胶片，凝胶成像灰度定量系统对蛋白条带进行灰度分析。

### 1.7 统计学分析

各组实验重复3次，数据取均数，实验数据用SPSS 13.0进行统计学处理，*P* < 0.05为差异有统计学意义。

## 2 结果

### 2.1 克隆形成实验拟合剂量生存曲线

图 1是用Excel软件拟合的H358细胞剂量生存曲线，以放疗剂量为横坐标，SF值取对数作为纵坐标，结果显示两组数据拟合的曲线分开，X线组各个剂量的SF值均大于X线+吉非替尼组，X线组SF2值为0.011, 1，D0值为4.435，Dq值为5.399，X线+吉非替尼组SF2值为0.000, 865，D0值为2.487，Dq值为2.494，SER值为2.815。

**1:**
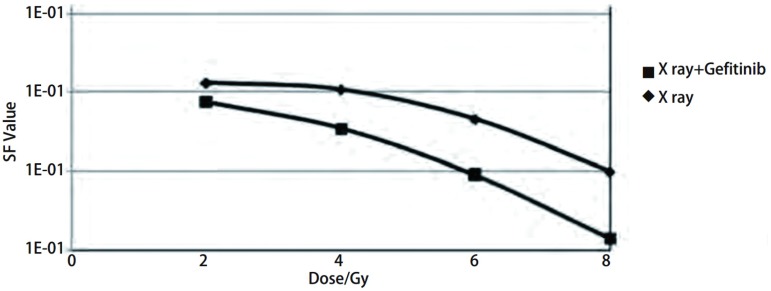
H358细胞剂量生存曲线 H358 cells dose-survival curve

### 2.2 激光共聚焦显微镜观察放疗后细胞核内γ-H2AX焦点情况

图 2为激光共聚焦显微镜照相显示H358细胞经4 Gy X照射线后不同时间点细胞核内γ-H2AX焦点的情况，蓝色底代表H358细胞核，红色光点即为γ-H2AX焦点。X线组放疗后10 min细胞核内开始出现γ-H2AX焦点并随时间增加，在60 min达高峰，随后递减（图 2A）; 而X线+吉非替尼组的γ-H2AX焦点数在各时间点均比X线组多，而且一直持续到放疗后12 h细胞核内还有较多γ-H2AX焦点，24 h仍存在（图 2B）。

**2:**
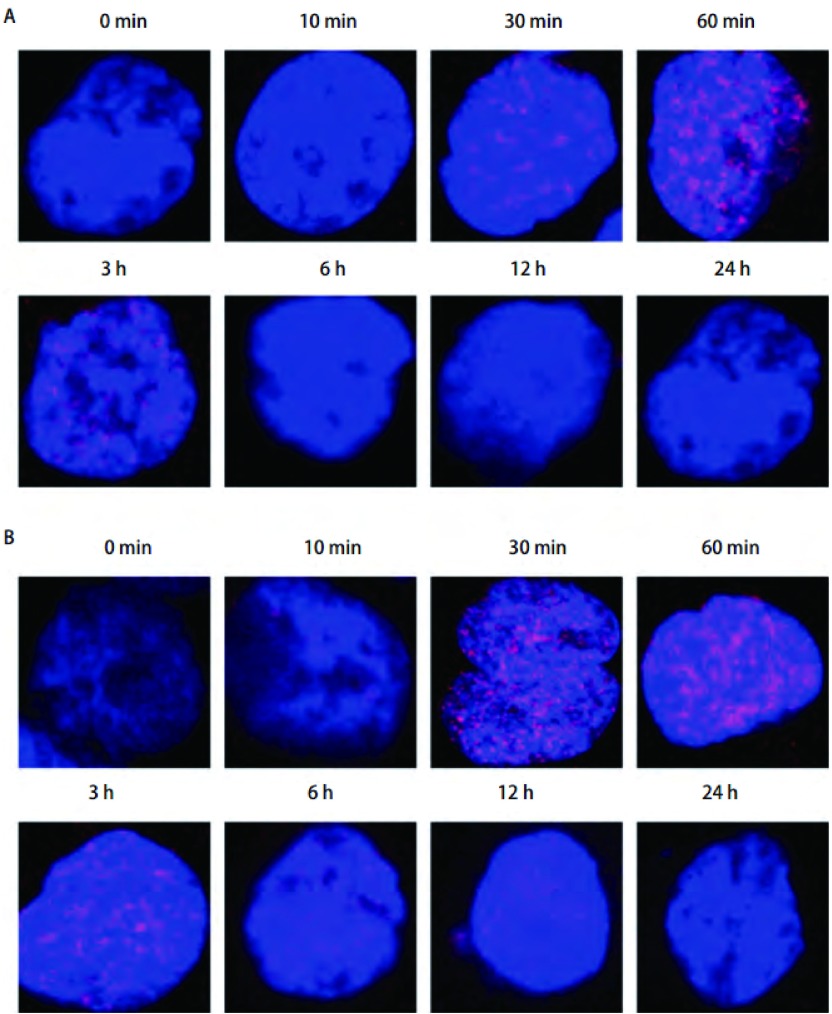
激光共聚焦显微镜观察H358细胞4 Gy放疗后不同时间点细胞核内γ-H2AX焦点的情况。A：X线组; B：X线+吉非替尼组。 Immunostaining for confocal microscopy testing for nuclear γ-H2AX foci of H358 cells after 4 Gy X ray. A: X ray group; B: X ray+Gefitinib group.

### 2.3 激光共聚焦显微镜观察放疗后EGFR入核情况

激光共聚焦显微镜观察照相显示H358细胞4 Gy放疗后不同时间点细胞核内EGFR表达情况，蓝色代表H358细胞核，红色光点为EGFR，X线组0 min时EGFR焦点位于胞浆或核周，在放疗后进入细胞核内，45 min达高峰，后逐渐减少（图 3A）; 而在X线+吉非替尼组，EGFR焦点分布在核外胞浆中，没有单纯放疗组的入核趋势（图 3B）。

**3:**
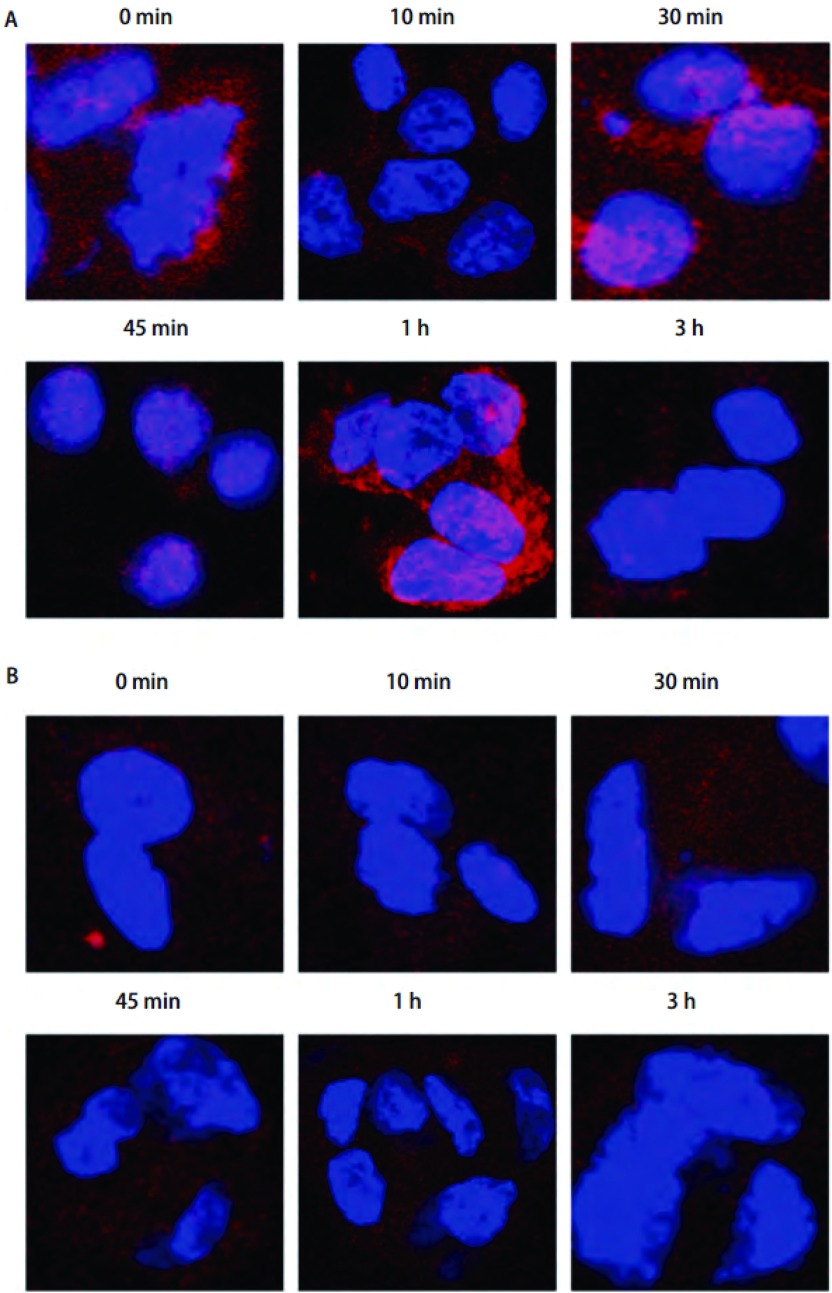
激光共聚焦显微镜观察H358细胞4 Gy放疗后不同时间点细胞核内EGFR焦点的情况。A：X线组B：X线+吉非替尼组。 Immunostaining for confocal microscopy testing for nuclear EGFR foci of H358 cells after 4 Gy X ray. A: X ray group; B: X ray+Gefitinib group.

### 2.4 Western blot检测细胞核内EGFR表达及蛋白条带灰度定量分析

图 4为Western blot检测H358细胞放疗后1 h内各时间点核蛋白EGFR的表达，X线组在放疗后15 min细胞核内即有EGFR表达，且条带随时间而加深（图 4A）; 而X线+吉非替尼组无EGFR核蛋白条带（图 4B）。根据蛋白条带采用凝胶成像灰度定量系统计算各条带的灰度值，并绘制成柱状图（图 4C），X线组的灰度值均高于X线+吉非替尼组（*P*=0.042）。说明H358细胞在放疗后1 h内，在各个相同的时间点，X线+吉非替尼组细胞核EGFR表达少于X线组，可能是吉非替尼通过某种途径阻止EGFR放疗后进核。

**4:**
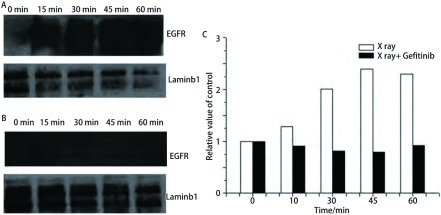
Western blot检测H358细胞4 Gy放疗后不同时间点细胞核内EGFR的表达情况。A：X线组; B：X线+吉非替尼组; C：EGFR带灰度定量柱状图。 Western blot testing for nuclear EGFR of H358 cells after 4 Gy X ray. A: X ray group; B: X ray+Gefitinib group; C: The densitometric analysis of EGFR expression.

## 3 讨论

ErbB家族由EGFR（erbB1）、erbB2（Neu）、erbB3和erbB4四个成员组成，属于跨膜糖蛋白。配体结合导致胞内EGFR的二聚体化，进而激活受体及下游的各种信号转导通路，如Ras/Raf/MEK/ERK、PKC、STATs和PI3K/Akt途径^[7, 8]^。在肿瘤细胞，EGFR激活引起的信号通路主要调节肿瘤细胞增殖、分化、细胞存活、细胞周期进展及血管生成^[9]^。肿瘤细胞EGFR及下游信号通路的激活有以下途径：①配体与EGFR过表达的肿瘤细胞结合; ②配体非依赖的途径：*EGFR*突变或EGFR下游信号分子突变，如RAS或PTEN^[10, 11]^。另外，放疗也可导致EGFR及其下游信号通路的激活。这些途径的激活在肿瘤治疗引起的化疗及放疗耐受中起着重要作用^[12, 13]^。

放疗是NSCLC治疗的重要组成部分，真核生物中，放疗所致的DSB修复有两种方式：同源重组（homologous recobination, HR）和非同源断端连接（non-homologous ends jion, NHEJ），其中NHEJ在放疗导致的DSB修复中占主导地位^[14]^。NHEJ由DNA依赖性蛋白酶（DNA-PK）、XRCC4、DNA连接酶IV及ATM催化，与Mre11、Rad50和Nbs1（MRN）形成复合物从而使DSB断端再连接而修复^[15]^。但是除了SCLC与NSCLC之间敏感性的差异，同一组织类型的敏感性差异也存在。研究^[16]^显示，EGFR是决定放疗敏感性的重要因素之一，野生型EGFR可以在放疗后入核，与DNA-PKcs结合，从而对DSB进行修复，因而对放疗抵抗; 而突变型EGFR则不能入核与DNA-PKcs结合，所以对放疗相对敏感。基于EGFR在肿瘤放疗修复中的作用，分子靶向药物小分子酪氨酸激酶受体抑制剂（tyrosine kinase recepter inhibitor, TKI）及作用于EGFR的单克隆抗体被单药或与化/放疗联合用于临床。西妥昔单抗（C225）与放疗联合用于头颈部肿瘤，患者的总生存率明显比单纯放疗组高。这可能基于西妥协单抗对肿瘤细胞的放疗增敏作用，这已在临床前的研究^[17, 18]^中证实。研究^[17]^显示，C225是通过抑制放疗引起的EGFR入核，废除EGFR-DNA-PKcs的相互作用，从而延迟DNA损伤修复，起到头颈部肿瘤放疗增敏的作用。吉非替尼是小分子受体抑制剂，它通过与EGFR酪氨酸激酶结合而起作用，本实验旨在探讨吉非替尼是否与C225相似有对NSCLC有放疗增敏的作用以及可能机制。

本课题选取H358细胞株，它是EGFR野生型人NSCLC细胞株，EGFR在基因水平上扩增，对吉非替尼敏感。选择1 μg/mL吉非替尼作为增敏浓度，是根据吉非替尼的每日处方剂量后测患者的血药浓度及H358的IC_20_而定^[16]^。吉非替尼的半衰期为24 h。

克隆形成实验证明吉非替尼可提高人NSCLC细胞株H358的放疗敏感性。从20世纪60年代，人们开始用离体细胞存活曲线来评价细胞放射的效应，因其可以反应细胞增殖性死亡，故仍最适于反应细胞照射后存活情况。本实验根据H358细胞放疗后克隆形成情况，计算SF值绘制剂量生存曲线，可以看到X线+吉非替尼组各个剂量的细胞存活率均低于X线组，SF2值也同样如此。且X线+吉非替尼组的D0、Dq也低于X线组，SER为2.185。说明前者放疗后发生增殖性死亡的细胞多于后者，大部分细胞因丧失了继续增殖生长的能力而不能形成克隆，即吉非替尼使H358细胞的放疗敏感性增加。

实验还显示，吉非替尼增加放疗引起的H358细胞核内γ-H2AX的表达，并延长其存在时间。在真核细胞中，外源性化学、物理及生物性物质所致的DSB都可能会触发H2AX的快速磷酸化，从而形成γ-H2AX。可用免疫荧光技术，清楚地观测到以γ-H2AX焦点形成为特征的DNA双链断裂的区域，这种染色质的改变通常被认为是细胞对DSB做出的敏感反应。γ-H2AX的形成在DNA的DSB中不仅起到损伤的标志性作用，而且在DNA的DSB修复中具有连接的作用^[19]^。实验中，X线+吉非替尼组放疗后细胞核内γ-H2AX焦点在各个时间点均多于X线组，焦点持续的时间也更长，说明吉非替尼增加了放疗引起的DSB形成，使H358细胞DNA损伤增加，并使DNA修复延迟。结合之前实验结果，吉非替尼通过影响放射后H358细胞的损伤及修复而提高放疗的的敏感性。

另外，通过免疫荧光激光共聚焦显微镜观察显示放疗后H358细胞核内EGFR的变化，结果是X线组EGFR在放疗后1 h内进入细胞核，而X线+吉非替尼组各组EGFR光点位于细胞浆及核周。与共聚焦结果相符，Western blot实验证明，X线组H358细胞放疗后1 h内EGFR表达随时间增加，而X线+吉非替尼组细胞核内没有EGFR表达，两组结果有统计学意义（*P* < 0.05）。由于EGFR可通过放疗激活进行下游信号通路的传导，其中野生型EGFR可直接在放疗后入核，与DNA-PKcs结合进行DSB修复起到放疗保护的作用，结合实验结果，我们可以推测，吉非替尼是通过抑制EGFR放疗后入核进行DSB修复，而增加H358细胞放疗敏感性。

肿瘤细胞的EGFR在放疗后迅速入核，*EGFR*基因敲除的细胞因DNA修复被抑制而显示出明显的放疗增敏作用^[20]^，这说明在细胞受到放射线后EGFR在肿瘤细胞的存活中起着重要作用，核内EGFR与DNA损伤的修复有着必然的联系。本实验的结果显示，EGFR是影响H358细胞放疗敏感性的重要因素，尤其是野生型EGFR高表达NSCLC，而吉非替尼能够通过与EGFR结合，使EGFR不能进入核内进行放疗后损伤的修复，从而使放疗敏感性增加。也有研究^[21]^显示，吉非替尼可通过抑制ATM的活性从而制止放疗引起的DNA损伤修复及诱使有丝分裂停止使细胞死亡而起到放疗增敏的作用。

然而，吉非替尼是否还通过其它途径影响H358细胞放疗敏感性; 入核后是否通过影响EGFR-DNA-PKcs复合物的形成而起作用，抑制DNA-PKcs的活性是否会对核内EGFR的表达及放疗敏感性产生影响，后续的研究正在进行; 体内实验与体外实验是否能得出相同的结论; 能否将EGFR作为放疗增敏的靶点应用于临床，以上问题还需要进一步的体外及体内实验来验证。
